# Sabiporide Reduces Ischemia-Induced Arrhythmias and Myocardial Infarction and Attenuates ERK Phosphorylation and iNOS Induction in Rats

**DOI:** 10.1155/2013/504320

**Published:** 2012-12-30

**Authors:** Henri Doods, Dongmei Wu

**Affiliations:** ^1^CNS Diseases Research, Boehringer Ingelheim Pharma KG, D-88397 Biberach, Germany; ^2^Department of Research, Mount Sinai Medical Center, Miami Beach, FL 33140, USA; ^3^WCU Program, Department of BIN Fusion Technology, Chonbuk National University, Jeonju 561-756, Republic of Korea

## Abstract

The aim of the present study was to investigate the effects of sabiporide, a potent and selective NHE1 inhibitor, on myocardial ischemia-induced arrhythmias and myocardial infarction and the possible pathways related to the cardioprotection afforded by sabiporide treatment. Anesthetized rats were subjected to myocardial ischemia via left main coronary artery occlusion for 30 minutes, followed by 2 hours of reperfusion. Administration of sabiporide (0.01–3.0 mg/kg) prior to coronary artery occlusion dose-dependently reduced ischemia-induced arrhythmias and infarct size with an ED50 value of 0.14 mg/kg. Administration of sabiporide (1.0 mg/kg) prior to reperfusion also reduced infarct size by 38.6%. The reduction in infarct size was accompanied by a decrease in circulating levels of creatine phosphokinase and troponin I. In addition, sabiporide (1.0 mg/kg) given prior to coronary artery occlusion or immediately before reperfusion significantly reduced phosphorylation of the extracellular signal-regulated kinase (ERK1/2) and the expression of the inducible nitric oxide synthase (iNOS) following myocardial ischemia-reperfusion. This study demonstrates that sabiporide is a potent and effective cardioprotective agent during myocardial ischemia and reperfusion, by reducing serious ventricular arrhythmias and myocardial infarct size. The cardioprotection afforded by sabiporide is attributed in part to inhibition of ERK1/2 phosphorylation and suppression of iNOS expression.

## 1. Introduction

Na^+^/H^+^ exchangers (NHEs) are membrane proteins that regulate ion fluxes. Physiologically, they extrude one intracellular proton in exchange for one extracellular sodium, thereby regulating intracellular pH. NHE1, the housekeeping isoform present in all mammalian cells, is the most predominant isoform in cardiomyocytes [1, 2]. It is implicated in heart hypertrophy and heart failure and mediates myocardial damage that occurs after ischemia-reperfusion injury. For this reason, regulation of NHE1 has been proposed as a therapeutic target for cardioprotection [1–3].

Alterations in energy metabolism during acute ischemia and reperfusion cause disturbances in the ion homeostasis of myocardial cells. Intracellular acidosis is the major stimulus that regulates NHE1 activity. The reduction in intracellular pH during ischemia due to anaerobic metabolism and ATP hydrolysis stimulates Na^+^/H^+^ exchange leading to increased sodium influx and elevation in intracellular calcium concentration through increased Na^+^/Ca^2+^ exchange, resulting in cellular injury [1–3]. In addition to activation of NHE1 during ischemia-reperfusion as a result of proton-dependent processes, a variety of endogenous mediators and oxidant stress produced by ischemia-reperfusion act to stimulate phosphorylation of the NHE1 cytosolic domain. These agents shift the set point of the antiporter such that it remains active in a more alkaline pH range [4, 5]. Inhibition of NHE1 has been shown to provide marked cardioprotection in a number of *in vitro* and *in vivo* models [1, 2].

Sabiporide, a benzoguanidine, is a potent selective inhibitor of NHE1. Considering its ability to inhibit initial rates of ^22^Na^+^ uptake, sabiporide is considered as one of the best NHE-1 inhibitors (Ki of 5 ± 1.2 10^-8 ^M). Furthermore, it discriminated efficiently between the NHE1, 2, and 3 isoforms (Ki for NHE2: 3 ± 0.9 10^-6 ^M and Ki > 1 mM for NHE3), and rinse-out kinetics showed that inhibition with sabiporide is extremely persistent as compared to amiloride and cariporide (half time of 7 hours for sabiporide and of 1 and 2.5 minutes for amiloride and cariporide, resp.) [6–8]. Our recent study showed that postresuscitation pharmacological conditioning with sabiporide afforded protection from whole body ischemia-reperfusion injury by improving cardiac function, enhancing blood flows to vital organs and attenuating systemic proinflammatory response in an experimental model of asphyxia-induced cardiac arrest in piglets [9]. However, the efficacy of sabiporide has not been examined in an animal model of regional ischemia-reperfusion injury. The present study investigated the dose-response effects of sabiporide on myocardial ischemia-induced arrhythmias and myocardial infarction in anesthetized rats and examined the potential signaling pathways underlying this protective response.

## 2. Methods

 The Wistar rats (Chbb: Thom 350–380 g) used in this study received humane care in compliance with the “Guide for the Care and Use of Laboratory Animals” published by the US National Institutes of Health (NIH Publication no. 85-23, revised 1996). Performance of this project was granted approval by the local IACUC Review Board. The NHE1 selective inhibitor, sabiporide ((N-(aminoiminomethyl)-4-[4-(1H-pyrrol-2-ylcarbonyl)-1-piperazinyl]-3-(trifluoromethyl)-benzamide) was synthesized by Boehringer Ingelheim Pharma KG, Ingelheim, Germany [6].

### 2.1. Ischemia-Reperfusion Protocol

Rats were anesthetized with sodium pentobarbitone: initial induction at the 60 mg/kg dose (intraperitoneally [i.p.]) followed by continuous infusion at the dose of 30 mg/kg/h (subcutaneously [s.c.] in the abdominal skin through a 23G needle using a solution of 10 mg/mL) throughout the experiment. The animals were maintained in a deep surgical plane of anesthesia throughout the experiment by continuous blood pressure monitor and using the pin-prick of the hind leg method to determine the degree of the anesthesia. The trachea was cannulated, and the animals were artificially ventilated (80 strokes/min) with room air supplemented with oxygen. The body temperature was maintained at 37°C with a heating pad. The right carotid artery and left jugular vein were cannulated for continuous measurement of arterial blood pressure and intravenous administration of test agents (or vehicle: saline), respectively. Heart rate was derived from the blood pressure signal. 

A left-sided thoracotomy was performed at the level of the fifth intercostal space. A 5-0 silk suture was placed around the left main coronary artery approximately 1-2 mm from its origin. Four pieces of number 16 sewing cotton were coligated along with the coronary artery to facilitate reperfusion. The coronary artery was occluded for 30 minutes followed by 2 hours of reperfusion. Reperfusion was instituted by removing the ligature. Blood pressure and heart rate were measured continuously throughout the experiment. 

A lead II electrocardiogram (ECG) was recorded on a computer by Chart V3.5 program. Arrhythmias were evaluated according to the guidelines of the Lambeth conventions [10]. Ventricular premature beats (VPBs) were defined as discrete and identifiable premature QRS complexes (premature in relation to the P wave). All the VPBs occurring in the ischemic period (30 minutes) were counted. Ventricular tachycardia (VT) was defined as a run of four or more consecutive VPBs. The duration of VT, in seconds, was measured. Ventricular fibrillation (VF) was defined as a signal for which individual QRS deflections could no longer be distinguished from one another. The incidence of VF was quantitated in all rats.

At the end of the reperfusion period, the coronary artery was reoccluded. Evan's blue dye (1 mg/mL) was infused to the right ventricle to define the area of myocardium at risk. After this procedure, the heart was removed. Both atria and the roots of the great vessels were removed. The entire ventricle was cut from the apex to base into four transverse slices and incubated in 2,3,5-triphenyltetrazolium chloride (TTC) (10 mg/mL in phosphate buffer) for a period of 10 min at 37°C to visualize the infarct area. Each section was scanned by a color image scanner, and infarct size on the surface of each slice was determined by Photoshop 6.0 program. The infarct area were traced manually on the digital images and automatically measured. The infarct size was expressed as percentage of area at risk (AAR). The area at risk was similar between all animals, with an average of 62 ± 4% of LV. In some hearts (those which were not subjected to TTC staining), the free walls of the left ventricles (ischemia region or sham) were separated and snap-frozen for immunoanalysis. 

Saline or sabiporide (0.01, 0.1, 0.3, 1.0, and 3.0 mg/kg) was given 10 min before coronary artery occlusion; or sabiporide (1.0 mg/kg) was administered immediately before the start of reperfusion.

### 2.2. Measurement of Creatine Phosphokinase (CPK) Activity and Troponin I

Blood samples were removed from carotid artery before drug treatment, before reperfusion, and at the end of experiment. They were promptly centrifuged at 12,500 rpm, 4°C, for 15 min, the plasma removed, and stored at –80°C until assayed. Creatine phosphokinase levels were determined using a CPK kit (Sigma, Steinheim, Germany). Plasma troponin I levels were measured using a cardiac troponin I enzyme immunoassay kit (Life Diagnostics, Inc., West Cheater, PA, USA).

### 2.3. Western Blot Analysis

Rat left ventricle tissue lysate was prepared by homogenization in ice cold RIPA buffer. Tissue and cell debris were removed by centrifugation. Protein concentration was determined by the Bio-Rad protein assay. The protein extracts were boiled for 5 min in SDS loading buffer and loaded onto NuPAGE 4–12% Bis-Tris Gel (Invitrogen, Carlsbad, CA, USA). After electrophoresis, the separated proteins were transferred onto nitrocellulose membrane (Invitrogen, Carlsbad, CA, USA). The blots were incubated in 5% nonfat dry milk in Tris-buffered saline for 2 h at room temperature and incubated overnight at 4°C with primary antibody: extracellular signal regulated protein kinase (ERK1/2) (1: 500) (Abcam Inc., Cambridge, MA, USA), phospho-ERK1/2 (1: 2000); inducible nitric oxide synthase (iNOS) (1: 250); and GAPDH (1: 3000) (all from Santa Cruz Biotechnology, Santa Cruz, CA, USA). The blots were rinsed with Tris-buffered saline and incubated with HRP-conjugated donkey anti-rabbit or goat anti-mouse IgG secondary antibody for 2 hours. Immunoreactivity was detected using electrochemiluminescence autoradiography (ECL kit; Amersham, Piscataway, NJ, USA). The levels of the signals were detected by a color image scanner and quantified by densitometry with the use of the NIH Image J software. All bands were normalized against GAPDH. The intensity of the protein bands in the MI groups were expressed as percentage of the negative control tissue samples (from the sham animals involving an identical surgical procedure without MI). All the relative ratio values obtained from 6 rats/groups were pooled and presented as mean ± SEM. 

### 2.4. Statistical Analysis

Data were analyzed for significance using GraphPad InStat software. Differences between groups in hemodynamic and infarct sizes were compared using ANOVA (with Bonferroni posttest) for multiple comparisons. Comparisons for the number of VPBs and duration of VT during ischemia were analyzed with Mann-Whitney *U* test. Comparisons for the incidence of VF were analyzed with Fischer's exact probability test. The results are presented as means ± SEM. *P* values <0.05 were considered to be significant.

## 3. Results

### 3.1. Hemodynamic and Arrhythmia Data

**Figure 1:**
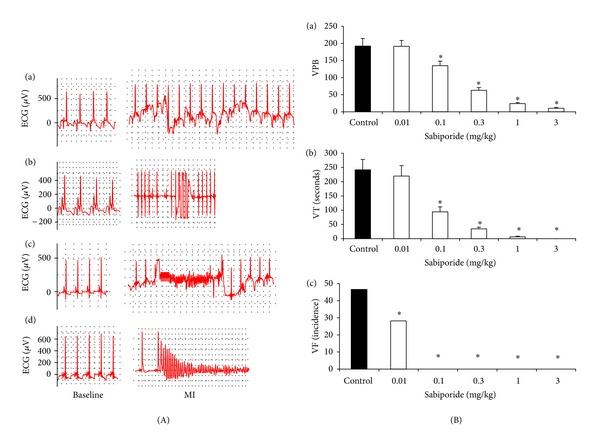
(A) Sample tracing EKG data from rats at baseline and myocardial ischemia (MI). (a) ventricular premature beats; (b) Ventricular tachycardia (VT); (c) ventricular fibrillation (VF) (reversible, animal survived); (d) ventricular fibrillation (irreversible, animal died). (B) Dose-dependent effects of sabiporide on (a) ventricular premature beats, (b) ventricular tachycardia duration, and (c) ventricular fibrillation incidence in anesthetized rats. All values are the mean ± SEM, *n* = 6. *P < 0.05 versus the vehicle control group.

Consistent with previous findings on the activities of NHE1 inhibitors, sabiporide has no direct effect on blood pressure and heart rate (Table 1). All control rats exhibited VPB; 15 of 17 rats showed VT, while 13 of 17 showed VF. In addition, 5 of 17 control rats died by VF-induced cardiac arrest during the 30-minute coronary artery occlusion period. However, administration of sabiporide prior to coronary artery occlusion dose-dependently reduced the number of VPB, the duration of VT, and the incidence of VF (Figures 1(a), 1(b), and 1(c)). VF and death was completely prevented by sabiporide at the doses ranging from 0.1 to 3.0 mg/kg. One out of 7 rats died at the doses of 0.01 and 0.03 mg/kg, respectively. 

### 3.2. Myocardial Infarction

**Figure 2:**
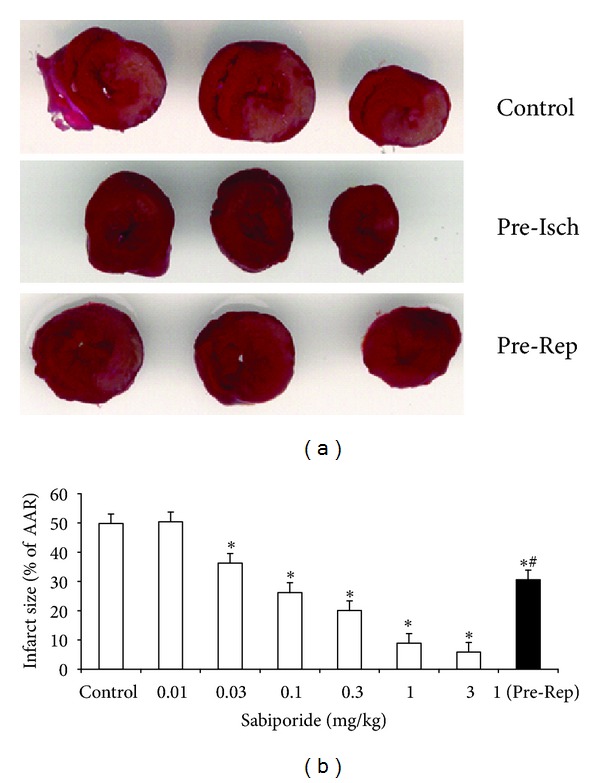
(a) Scanning graphs of the infarct area in control rats and those that received sabiporide (1 mg/kg) prior to ischemia (Pre-Isch) or reperfusion (Pre-Rep). (b) Dose-dependent effects of sabiporide in myocardial infarct size. Open column: sabiporide (0.01, 0.03, 0.1, 0.3, 1.0, and 3.0 mg/kg) or saline was administered intravenously 10 minutes before occlusion. Black column: sabiporide (1.0 mg/kg) was administered intravenously at the start of reperfusion. All values are the mean ± SEM, *n* = 6. *P < 0.05 versus the control group. ^#^ *P* < 0.05 versus sabiporide (1.0 mg/kg) prereperfusion group.

Occlusion of the left main coronary artery for 30 minutes followed by 2 hours of reperfusion resulted in substantial injury to the myocardium. In the control group, occlusion and reperfusion produced an infarction of 49.8% (IF/AAR) (Figure 2). Meanwhile, treatment of sabiporide prior to coronary artery occlusion dose-dependently reduced the infarct size (Figure 2). The ED50 value was found to be 0.14 mg/kg. Administration of sabiporide (1.0 mg/kg) prior to reperfusion also reduced infarct size by 39% (Figure 2).

### 3.3. The Release of CPK and Troponin I

**Figure 3:**
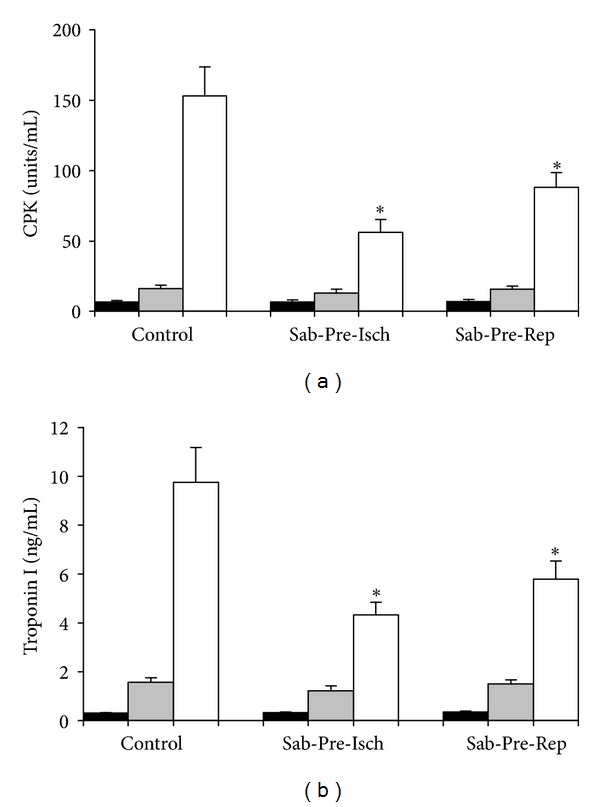
Effect of sabiporide (1 mg/kg) given prior to ischemia or prior to reperfusion on plasma levels of CPK (a) and troponin I (b) following myocardial ischemia-reperfusion on anesthetized rats. All values are the mean ± SEM, *n* = 6. *P < 0.05 versus the control group. Black column: baseline; grey column: before reperfusion; white column: at the end of experiment.

As shown in Figure 3, plasma levels of CPK and troponin I were significantly elevated after ischemia-reperfusion. Treatment with sabiporide reduced the plasma levels of CPK and troponin I by 63% and 55%, respectively. Sabiporide (1.0 mg/kg) given prior to reperfusion also reduced the plasma levels of CPK and troponin I by 42% and 40%, respectively.

### 3.4. Western Blot Analysis

**Figure 4:**
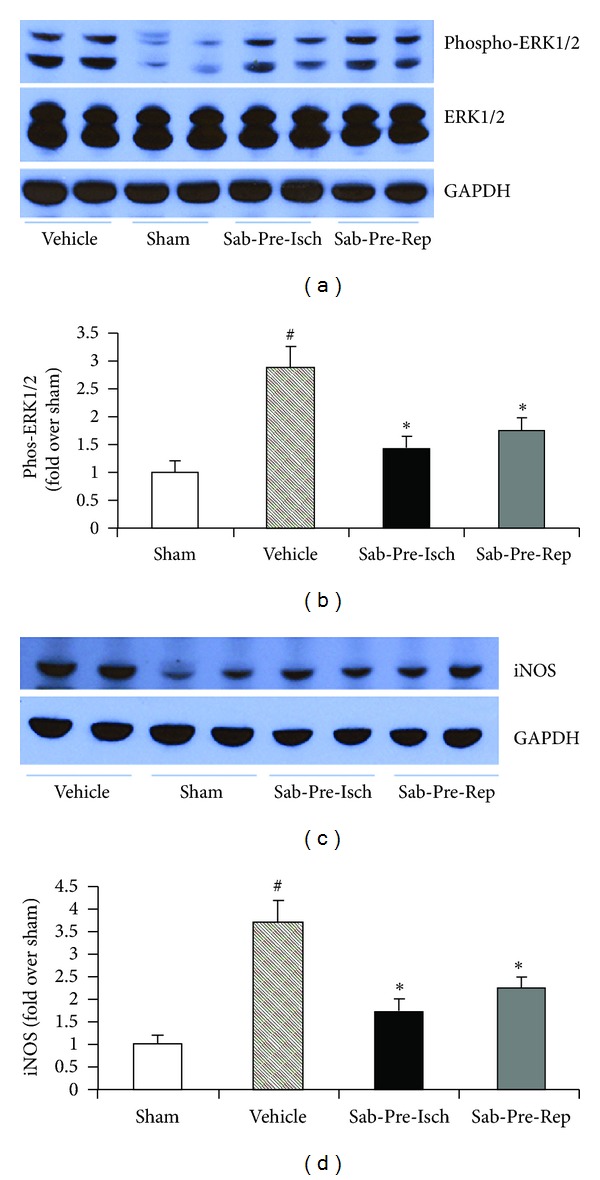
ERK1/2 expression and phosphorylation and iNOS expression in heart tissues from rats subjected to coronary ligation and reperfusion. Sabiporide (1.0 mg/kg) was given 10 min prior to coronary occlusion or immediately before reperfusion. (a) Representative blots of phosphorylated ERK1/2 and total ERK1/2 in heart tissues. (b) Statistical data obtained from quantitative densitometry of phos-ERK1/2 in heart tissues. (c) Representative blots of iNOS expression. (d) Statistical data obtained from quantitative densitometry of iNOS in heart tissues. Data are the mean ± SEM, *n* = 6. ^#^ *P* < 0.05 versus sham. *P < 0.05 versus the vehicle control group.

Activation of ERK1/2 and induction of iNOS are known to be involved in myocardial ischemia-reperfusion injury [11, 12]. To further identify the possible pathways underlying sabiporide-induced cardioprotective effects, we measured the ERK1/2 expression and phosphorylation and iNOS expression in myocardium at the end of reperfusion by western blotting (Figures 4(a), 4(b), 4(c), and 4(d)). No difference in total ERK1/2 expression was observed among different treatment groups. However, ischemia-reperfusion resulted in a significant increase in ERK1/2 phosphorylation and sabiporide (1.0 mg/kg) given prior to coronary artery occlusion or prior to reperfusion significantly reduced ERK1/2 phosphorylation following ischemia-reperfusion by 51% and 38%, respectively. Ischemia-reperfusion also resulted in significantly increased iNOS expression. Treatment of sabiporide (1.0 mg/kg) prior to coronary artery occlusion or prior to reperfusion significantly reduced iNOS expression in heart tissue following ischemia-reperfusion by 53% and 40%, respectively.

## 4. Discussion

The effects of sabiporide on ischemia arrhythmias, myocardial infarction, ERK1/2 phosphorylation, and iNOS expression were studied in a rat model of myocardial ischemia-reperfusion injury. Administration of sabiporide prior to ischemia resulted in a dose-dependent reduction of ischemia-induced arrhythmias, including ventricular tachycardia, and ventricular fibrillation as well as reduction of myocardial infarct size. In addition, treatment with sabiporide prior to coronary artery occlusion or prior to reperfusion significantly reduced ERK1/2 phosphorylation and cardiac iNOS expression following ischemia-reperfusion.

Coronary artery disease still remains the leading cause of death in the industrialized world despite considerable progress in its management. In patients with severe left ventricular dysfunction, more than 60% of deaths are attributed to the development of ventricular arrhythmias during periods of myocardial ischemia or infarction [13, 14]. The mechanisms of ventricular arrhythmias in acute myocardial ischemia and infarction have been mainly studied using animal models. A number of* in vivo *studies in rats have shown that acute myocardial ischemia with coronary artery occlusion results in severe ventricular arrhythmias [14–16]. Consistent with the findings of previous reports, the present study found that left main coronary artery occlusion resulted in serious ventricular arrhythmias, including ventricular premature beats, ventricular tachycardia and fibrillation, resulting in early death in rats. 

Acute myocardial ischemia is associated with significant intracellular and extracellular ionic and metabolic alterations of the myocardium. Some of extracellular changes include elevated potassium, increased lactate and carbon dioxide production, acidosis, and catecholamine release, which concomitantly produce intracellular acidosis, elevated concentrations of calcium, magnesium, and sodium ions [14, 17]. These biochemical and metabolic changes alter inward and outward transmembrane ionic current fluxes, causing profound alterations of the resting membrane and action of potential characteristics of the myocyte. Changes such as depolarization of the resting membrane potential, diminished upstroke velocity, slowed conduction, decreased excitability, shortening of the action potential duration, altered refractoriness, dispersion of repolarisation, and abnormal automaticity, can all occur [14]. The resultant biochemical and electrical changes do not all occur at once; however, intracellular Na^+^ and Ca^2+^ overload may be the final common pathway leading to cardiac arrhythmias following myocardial ischemia by activating the transient inward current or causing electrical uncoupling of cardiac myocytes [18, 19]. Thus, altered intracellular Na^+^ and Ca^2+^ resulting from pH-regulated NHE1 activation (see reviews [4, 5]) may be an important contributor to cardiac arrhythmias following cardiac ischemia and that blocking NHE1 could be effective in attenuating ischemia-induced cardiac arrhythmias. Consistent with the observation that NHE1 inhibition can reduce ventricular arrhythmias [20, 21], the present study found that NHE1 inhibition with sabiporide dose-dependently reduced ventricular premature beats and ventricular tachycardia and prevented ventricular fibrillation and early death. This indicates that sabiporide is a potent and effective agent to attenuate cardiac arrhythmias.

Reducing myocardial infarct size is the primary goal in patients with coronary heart disease. Early reperfusion of an occluded coronary artery is a well-known and effective strategy to reduce ischemia-induced myocardial damage [22, 23]. However, reperfusion itself has been shown to cause significant cardiac injury through several complex and unresolved mechanisms [24, 25]. It is well established that NHE1 activation during ischemia-reperfusion through pH-regulatory pathway and other pathways mediated by endogenous ischemia metabolites results in increased intracellular Na^+^ and Ca^2+^, leading to myocardium damage [1–3]. NHE1 inhibitors have been shown to protect the myocardium against ischemia-reperfusion damage [14, 15, 21]. In this study, administration of sabiporide prior to ischemia dose-dependently reduced the infarct size. Administration of 1.0 mg/kg sabiporide prior to reperfusion also reduced infarct size by 38.6%. The reduction in infarct size was accompanied by a reduction in CPK and troponin I release, indicating that ischemia-reperfusion- induced myocardium damage can be attenuated by NHE1 inhibition with sabiporide. 

Cardiac ischemia-reperfusion is associated with activation of various signaling pathways (e.g., extracellular signal regulated protein kinase (ERK1/2) pathway and inducible nitric oxide synthase (iNOS) mediated cellular injury pathway) that play an important pathophysiological role in the progression of ischemia-reperfusion injury and myocardial dysfunction [11, 12]. ERK1/2 participates in cellular signal transduction cascades and is activated by a diverse range of stimuli including oxidative stress, ischemia-reperfusion and vasoactive agents [11, 26–28]. Recent studies indicate a link between NHE1 activation and activation of ERK1/2 signaling pathways [29, 30]. In cardiac myocytes, sustained intracellular acidosis activates NHE1 and the ERK1/2 pathway [31]. Activation of ERK1/2 has been shown to stimulate NHE1 phosphorylation during acidosis and myocardial ischemia-reperfusion in neuron cultures, cardiac myocytes, and isolated hearts [29, 31]. However, the link between NHE1 and the ERK1/2 pathway in animal models of myocardial ischemia-reperfusion has not yet been identified. In the present study, we showed that pretreatment with sabiporide significantly reduced ERK1/2 phosphorylation following ischemia and reperfusion. This finding indicates that NHE1 activation may have directly triggered ERK1/2 phosphorylation, or indirectly by causing cellular injury which triggered oxidative stress and the release of vasoactive agents that induced ERK1/2 activation. Physical stress (ischemia-reperfusion) and some vasoactive agents (phenylephrine, endothelin-1, and angiotensin II) have been known to trigger activation of both NHE1 and ERK1/2 [1, 2, 29–31]. Therefore, further studies are needed to determine whether activation of NHE1 can directly or indirectly trigger ERK1/2 phosphorylation. 

Increased iNOS expression is a component of the immune response and has been demonstrated in cardiomyocytes in ischemia-reperfusion, septic shock, myocarditis, transplant rejection, dilated cardiomyopathy, and heart failure [32–34]. Studies indicate that nitric oxide produced by iNOS is cardiotoxic in that it suppresses myocardial contractility and increases myocyte apoptosis and mortality [33, 35, 36]. Cardiomyocyte overexpression of iNOS in mice results in peroxynitrite generation, heart block, and sudden death, suggesting a pathological role of iNOS induction in heart diseases [37]. Induction of iNOS expression is mediated through cytokine-inducible transcription factors, such as IFN regulatory factor-1 and nuclear factor kappa B (NF-*κ*B), to elements within the iNOS promoter, as well as through activation of nicotinamide adenine dinucleotide phosphate (NADPH) oxidase and reactive oxygen species (ROS) formation [38–41]. In a rabbit model of pacing-induced heart failure, Aker et al. [42] have shown that sabiporide significantly reduced myocardial apoptosis, fibrosis, myocyte cross-sectional area, p38MAPK phosphorylation, and iNOS protein expression. They have also shown that the progression of heart failure in rabbits is attributed in part to p38 MAP kinase activation and ROS formation [43]. We have previously reported that inhibition of NHE1 attenuates NF-*κ*B activation and reduces cytokines production in traumatic hemorrhage shock in pigs [44]. In the present study, treatment with sabiporide attenuated the induction of iNOS following ischemia-reperfusion. Thus, the findings from the present study suggest that in addition to blunting cellular ionic derangement through inhibition of pH-regulatory activation of NHE1 pathway, the salutary cardioprotection afforded by sabiporide may also in part be attributed to the inhibition of ERK1/2 phosphorylation and iNOS expression following ischemia-reperfusion. 

Collectively, the present study demonstrates that sabiporide is a very potent and effective agent for cardioprotection during myocardial ischemia and reperfusion by reducing serious ventricular arrhythmias and myocardial infarct size. The cardiac protection afforded by sabiporide is possibly in part attributed to inhibition of ERK1/2 phosphorylation and suppression of iNOS induction.

In addition to the detrimental role of NHE1 activation in acute cardiac injury, studies have also demonstrated that NHE1 activation contributes to chronic maladaptive myocardial responses to injury such as postinfarction myocardial remodeling, and likely contributes to the development heart failure [45]. Furthermore, NHE1 is ubiquitously expressed in all mammalian cells. Recent studies have also shown that NHE1 inhibition protects from multiorgan injury in conditions of whole body ischemic-reperfusion injury and global metabolic acidosis, including cardiac arrest and resuscitation, traumatic hemorrhagic shock, and sepsis [9, 46, 47]. Thus, NHE1 inhibitors offer substantial promise for clinical development for the treatment of acute myocardial injury and heart failure and could also have potential implications for whole body protection from systemic metabolic acidosis.

## Figures and Tables

**Table 1:** Hemodynamics in control and sabiporide-treated rats.

	Baseline	After 5 min drug treatment	Occlusion 30 min	Reperfusion	
				1 h	2 h
MBP (mmHg)					
Control	119.1 ± 6.5	114.2 ± 8.5	65.9 ± 3.9^#^	69.8 ± 4.5^#^	67.6 ± 5.1^#^
Sabiporide (0.01 mg/kg Pre-Isch)	115.6 ± 8.1	122.3 ± 7.6	70.1 ± 4.5^#^	67.3 ± 5.2^#^	70.4 ± 6.4^#^
Sabiporide (0.1 mg/kg Pre-Isch)	120.8 ± 9.3	118.9 ± 10.6	68.4 ± 5.3^#^	65.3 ± 5.3^#^	66.9 ± 5.6^#^
Sabiporide (1 mg/kg Pre-Isch)	113.4 ± 8.7	117.8 ± 7.9	68.5 ± 4.9^#^	69.2 ± 7.1^#^	67.3 ± 5.1^#^
Sabiporide (3 mg/kg Pre-Isch)	121.8 ± 7.9	120.3 ± 8.4	72.4 ± 6.1^#^	67.2 ± 5.8^#^	71.2 ± 6.6^#^
Sabiporide (1 mg/kg Pre-Rep)	114.5 ± 6.7	115.4 ± 6.8	66.1 ± 3.5^#^	64.5 ± 5.5^#^	71.3 ± 5.3^#^

HR (bpm)					
Control	428.1 ± 18.5	423.7 ± 25.1	323.0 ± 22.4^#^	337.3 ± 20.2^#^	330.1 ± 23.1^#^
Sabiporide (0.01 mg/kg Pre-Isch)	416.7 ± 20.5	423.6 ± 22.7	315.6 ± 20.3^#^	323.4 ± 19.7^#^	330.5 ± 23.5^#^
Sabiporide (0.1 mg/kg Pre-Isch)	423.7 ± 23.7	416.4 ± 19.9	324.9 ± 18.6^#^	321.7 ± 23.4^#^	319.7 ± 19.5^#^
Sabiporide (1 mg/kg Pre-Isch)	430.3 ± 21.8	425.7 ± 21.6	310.5 ± 18.4^#^	332.9 ± 21.7^#^	324.6 ± 17.8^#^
Sabiporide (3 mg/kg Pre-Isch)	411.7 ± 19.2	419.3 ± 22.5	304.7 ± 17.9^#^	326.8 ± 19.7^#^	321.8 ± 21.9^#^
Sabiporide (1 mg/kg Pre-Rep)	431.2 ± 20.7	420.2 ± 17.7	326.6 ± 24.3^#^	312.5 ± 18.5^#^	323.7 ± 21.2^#^

All values are the mean ± SEM, *n* = 6. ^#^ *P* < 0.05 versus the baseline values.
